# High resolution manometry as a diagnostic tool for gastro‐esophageal reflux: A step forward but not yet ready for clinical use

**DOI:** 10.1002/ueg2.12573

**Published:** 2024-04-18

**Authors:** Frank Zerbib

**Affiliations:** ^1^ Gastroenterology Department CHU de Bordeaux Centre Medico‐Chirurgical Magellan Hôpital Haut‐Lévêque Université de Bordeaux INSERM CIC 1401 Bordeaux France

The diagnosis of gastro‐esophageal reflux disease (GERD) is frequently clinical, based on the presence of troublesome typical symptoms, that is, heartburn, regurgitation, and chest pain. In this situation and in the absence of alarm symptoms, a pragmatic approach consisting of an empirical treatment with antisecretory medications is recommended.^1^ Despite a possible placebo effect, the response to antireflux medication is a pragmatic approach for the diagnosis of GERD in patients with typical symptoms. However, when predominant symptoms are not typical or if symptom relief is not achieved with anti‐reflux medications, the diagnosis of GERD needs further investigations.^2^ The last iteration of the Lyon consensus proposed several criteria for a definite diagnosis of GERD based on endoscopy and ambulatory reflux monitoring off and on therapy.^2^ Although the failure of the anti‐reflux barrier (hypotensive esophago‐gastric junction [EGJ], hiatal hernia) and ineffective esophageal motility (IEM) play a major role in GERD pathophysiology, parameters determined by esophageal high‐resolution manometry (HRM) appear only as adjunctive or supporting evidence for the diagnosis of GERD.^2^ In the past years, IEM,^3^ EGJ type,^4^ EGJ contractile integral (EGJ‐CI)^5^ as well as the straight leg raise (SLR) maneuver^6^ have been shown to be associated with GERD, the last three reflecting a defective anti‐reflux barrier.^7^ As an example, a positive SLR maneuver defined by an increase of intra‐esophageal pressure of more than 11 mmHg could predict pathological reflux with a sensitivity of 79% and a specificity of 85%.^6^ In this issue of the UEG Journal, Siboni et al. report the performance of a score combining these metrics to predict the risk of GERD, the so‐called Milan score.^8^ This score was first built according to the results obtained in a calibration cohort of 295 patients and then validated in a second cohort of 233 patients. The final normogram to calculate the Milan score included EGJ type (1, 2, or 3), the result of the SLR maneuver (below or above 11 mmHg), the value of EGJ‐CI and the presence of IEM. The main result reported by Siboni et al. is that 92.4% of patients with a score below 60 had a negative reflux study while 88% of those with a score above 210 had an acid exposure time (AET) above 6%. These results are indeed very interesting, and the authors should be commended for their efforts to build this model from the data obtained in a large cohort of patients. However, whether HRM results alone, based on the Milan score, could be enough for the diagnosis of GERD is debatable.

For the patients, the main benefit of this approach would be to avoid ambulatory reflux monitoring, especially catheter‐based pH and pH‐impedance recordings, which may be poorly tolerated. The issue of tolerability of catheter‐based recordings should be overcome by using wireless pH capsule as advocated by the Lyon consensus.^2^ Indeed, wireless pH monitoring not only allows prolonged recordings with a higher diagnostic yield^9^ but is also better tolerated than catheter‐based recordings.^10^ However, these devices are not available or affordable worldwide.

In clinical practice, for the time being, it seems difficult to avoid ambulatory reflux monitoring in patients with a high Milan score and therefore a high risk of GERD in the absence of outcome studies, that is, data on the ability of the score to predict the response to therapy. On the other hand, when the score is low, the probability of having an AET >6% is low but reflux monitoring remains essential to identify patients who may have inconclusive AET values (between 4% and 6%) and more importantly to phenotype patients who may have functional esophageal disorders such as reflux hypersensitivity.^11^

There is no doubt that the Milan score represents a step forward and that further studies will determine how helpful it is for the management of patients with reflux like symptoms. Including simple clinical variables to the score such as typical versus atypical symptoms, BMI, and response to antisecretory therapy may increase the relevance of HRM parameters to determine the risk of having GERD. Another approach may be to perform a postprandial high‐resolution impedance manometry (HRiM) which allows not only to calculate the Milan score but also to identify the mechanisms of reflux episodes in the postprandial period (actual reflux, rumination, or supragastric belches).^12^ Studies are ongoing to determine how postprandial HRiM compares with ambulatory reflux monitoring. Maybe another step forward…

## CONFLICT OF INTEREST STATEMENT

Consultancy for Reckitt Benckiser, Medtronic.
